# Seasonal Variations and Indoor–Outdoor Characteristics of Fluorescent Aerosol Particles in Japanese Office Buildings

**DOI:** 10.3390/bios16070380

**Published:** 2026-07-11

**Authors:** Shota Tsuchiya, U. Yanagi, Hoon Kim, Kei Shimonosono, Naoki Kagi

**Affiliations:** 1 Graduate School of Engineering, Kogakuin University, Tokyo 163-8677, Japan; dm25099@g.kogakuin.jp; 2 School of Architecture, Kogakuin University, Tokyo 163-8677, Japan; 3 School of Architecture, Shibaura Institute of Technology, Tokyo 135-8548, Japan; kimhoon@shibaura-it.ac.jp; 4 Department of Built Environment for Health, National Institute of Public Health, Saitama 351-0197, Japan; shimonosono.k.aa@niph.go.jp; 5 School of Environment and Society, Institute of Science Tokyo, Tokyo 152-8550, Japan; kagi.n.dc49@m.isct.ac.jp

**Keywords:** fluorescent aerosol particles (FAPs), bioaerosols, office buildings, indoor–outdoor comparison, size-resolved FAP fraction, particle size distribution

## Abstract

Fluorescent aerosol particles (FAPs) are widely used as a real-time proxy for primary biological aerosol particles; however, their seasonal characteristics and size-resolved distributions in office environments remain poorly understood. In this study, FAPs were measured in ten office spaces located in four distinct regions of Japan during summer and winter using a real-time Bioaerosol Sensor. Indoor and outdoor FAP concentrations, indoor/outdoor ratios, and the size-resolved FAP fraction were evaluated. Indoor FAP concentrations were generally below 100 particles per liter (p/L), although peak concentrations of 140 p/L in summer and 195 p/L in winter were observed. Significant seasonal differences were detected in most offices, with several buildings showing higher concentrations in winter. Many offices exhibited relative humidity levels below 40% during winter, suggesting that dry indoor conditions may have promoted particle resuspension and contributed to elevated FAP concentrations. Indoor–outdoor comparisons suggested contributions from both indoor sources and outdoor infiltration. The size-resolved FAP fraction increased markedly with particle size, with median indoor values reaching 40–74% for 2.0–5.0 μm particles and 96–100% for particles > 5.0 μm. These findings indicate that FAPs in office environments are strongly associated with coarse particles and exhibit substantial seasonal and building-dependent variability.

## 1. Introduction

In recent years, biological aerosol particles in indoor environments have attracted increasing attention because of their potential impacts on indoor air quality (IAQ), human health, and environmental processes. Primary biological aerosol particles (PBAPs) consist of particles of biological origin, including bacteria, fungi, pollen, viruses, as well as their fragments and metabolic by-products. PBAPs are recognized as important contributors to respiratory exposure, allergic reactions, the transmission of infectious diseases, and atmospheric processes such as cloud formation and climate interactions [1,2,3,4].

Since people spend more than 90% of their daily lives indoors [5], exposure to indoor bioaerosols is considered an important environmental health issue [6]. Previous studies have reported associations between indoor bioaerosols and allergic diseases, asthma, respiratory symptoms, and microbial infections [7,8,9,10]. Furthermore, following the coronavirus disease 2019 (COVID-19) pandemic, interest in aerosol transport and exposure through indoor air has increased substantially [11,12,13,14].

Indoor bioaerosols originate from various sources, including occupants, pets, plants, heating, ventilation, and air-conditioning (HVAC) systems, water supply and drainage systems, damp surfaces, resuspension of floor dust, and infiltration of outdoor air [15,16,17,18]. Among these factors, human occupancy and activities are considered major determinants of indoor bioaerosol dynamics. Bhangar et al. demonstrated that human activities increased emissions of fluorescent biological aerosol particles (FBAPs) by 5–69 times compared with quiescent conditions and reported that floor dust resuspension accounted for approximately 60–70% of FBAP emissions [19]. Similarly, Xu et al. found that fluorescent particle concentrations increased significantly with occupant density in classrooms [20].

Outdoor environmental conditions also strongly influence indoor aerosol characteristics. Adams et al. reported that microbial community compositions in mechanically ventilated office buildings closely reflected those of outdoor environments [21]. In addition, environmental factors such as air temperature, relative humidity (RH), precipitation, and wind speed are known to affect PBAP abundance [22,23,24,25,26]. These environmental factors can also influence microbial community composition [21,24,26]. Environmental conditions have also been reported to influence microbial community composition in outdoor air. Using a Wideband Integrated Bioaerosol Spectrometer (WIBS), Ma et al. observed elevated nighttime concentrations of fluorescent particles under conditions of high RH in an industrial area [27].

Traditionally, culture-based methods have been widely used to assess airborne microorganisms. Although culture-based methods remain the standard approach, they require post-sampling incubation and may underestimate viable but non-culturable (VBNC) microorganisms [28,29]. To overcome these limitations, increasing attention has been directed toward real-time optical sensing technologies based on laser-induced fluorescence (LIF) [4,13]. LIF instruments detect intrinsic fluorophores such as tryptophan, nicotinamide adenine dinucleotide (NAD(P)H), and flavins (e.g., riboflavin), enabling real-time characterization of individual particles [30]. Instruments including the Ultraviolet Aerodynamic Particle Sizer (UV-APS), WIBS, Spectral Intensity Bioaerosol Sensor (SIBS), and the recently developed Bioaerosol Sensor (BAS) have been applied for environmental monitoring [31,32,33,34]. These technologies have significantly improved our understanding of temporal variations, size distributions, and indoor–outdoor transport mechanisms of FAPs. Recent comparative evaluations of fluorescence-based bioaerosol instruments have further clarified the performance characteristics, advantages, and limitations of different real-time monitoring technologies under environmental conditions [9]. Recent long-term field observations have shown that fluorescence-based instruments can capture pronounced temporal variability of fluorescent aerosol particles under diverse environmental conditions, highlighting the value of continuous real-time monitoring [35].

Nevertheless, caution is required when interpreting fluorescence measurements. Previous studies have shown that non-biological particles, such as mineral dust, diesel soot, brown carbon, and textile fibers, can also produce fluorescent signals [30,36]. Therefore, FAPs detected by LIF instruments should be regarded as proxy indicators of PBAPs rather than direct measurements of biological particles [2,30].

Although real-time fluorescence-based aerosol measurements have increasingly been conducted in outdoor environments, residential buildings, and educational facilities [6,20,27,37,38], studies focusing on office environments remain limited, particularly studies simultaneously investigating seasonal variations, indoor–outdoor relationships, and size-resolved FAP fraction characteristics in mechanically ventilated office buildings. Moreover, quantitative information on size-resolved FAP concentrations and the proportion of size-resolved FAP fraction in office buildings remains limited.

Therefore, the objective of this study was to investigate real-time measurements of fluorescent aerosol particles using a BAS in multiple office buildings across Japan during summer and winter. Indoor and outdoor FAP concentrations, size-resolved FAP fraction, and seasonal characteristics were analyzed to clarify the characteristics of FAPs in office environments.

## 2. Materials and Methods

### 2.1. Office Buildings Investigated

Table 1 summarizes the characteristics of the investigated office buildings and their air-conditioning systems. The study sites were located in Miyazaki City, Miyazaki Prefecture, in the Kyushu region of southern Japan, as well as in Chiba, Kanagawa, and Saitama Prefectures in the Tokyo metropolitan area. Measurements were conducted during summer (August 2025) and winter (February 2026). A total of ten office spaces in seven office buildings were investigated. Buildings A–E were measured during the summer campaign, while Buildings A–F were included in the winter campaign. Buildings E and F are adjacent buildings located on the same site. In addition, the number of occupants in each surveyed office space during the measurements ranged from 15 to 40.

### 2.2. Measurement Parameters and Procedures

FAPs were measured using a BAS (KANOMAX, Osaka, Japan). Continuous measurements were conducted at 1-min intervals for approximately 30 min in each office space. The BAS uses a 450 nm blue laser diode as the excitation light source. Fluorescence emitted at wavelengths longer than the excitation wavelength is detected by a high-sensitivity avalanche photodiode (APD) through a high-pass optical filter. This optical configuration enables the detection of particles containing intrinsic fluorophores, such as flavins (e.g., FMN), while minimizing interference from elastically scattered excitation light. Because the BAS employs a single excitation wavelength and broadband fluorescence detection, the measured fluorescent aerosol particles (FAPs) represent particles exhibiting fluorescence under 450 nm excitation and may include both biological particles and a limited fraction of fluorescent non-biological interferents. Detailed information regarding the measurement principle and performance of the BAS has been reported previously by Yanagi et al. [34]. Following the indoor measurements, FAP concentrations were also measured outdoors at the same building.

Indoor air temperature, relative humidity (RH), and carbon dioxide (CO_2_) concentrations were continuously monitored at 5-min intervals throughout the year using a TR-76Ui temperature–humidity–CO_2_ data logger (T&D Corporation, Nagano, Japan). To characterize the thermal environment and ventilation conditions of each office, data collected during working hours (09:00–17:00) over the one-week period preceding the FAP measurements were obtained and analyzed.

### 2.3. Statistical Analysis

Differences in FAP concentrations between indoor and outdoor environments and between seasons were evaluated using the Wilcoxon signed-rank test. Differences among particle-size categories were assessed using the Mann–Whitney U test. Statistical analyses were performed using IBM SPSS Statistics Version 29 (IBM Corp., Armonk, NY, USA), and statistical significance was defined as *p* < 0.05.

## 3. Results

### 3.1. Indoor Temperature, Relative Humidity, and CO_2_ Concentration

**Figure 1:**
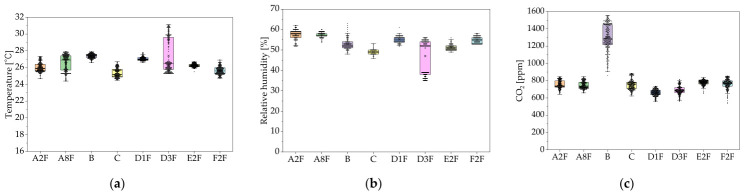
Indoor temperature (**a**), relative humidity (**b**), and CO_2_ concentration (**c**) in each investigated office space during summer. The labels shown in the figures represent the investigated buildings and locations; for example, A2F denotes the second floor of Building A, as described in Table 1. Note: In the box plots, the horizontal line represents the median, the × symbol represents the mean, the box represents the interquartile range (25th–75th percentiles), the whiskers indicate the minimum and maximum values excluding outliers, and the points indicate outliers. The same definitions apply to all subsequent figures containing box plots.

**Figure 2:**
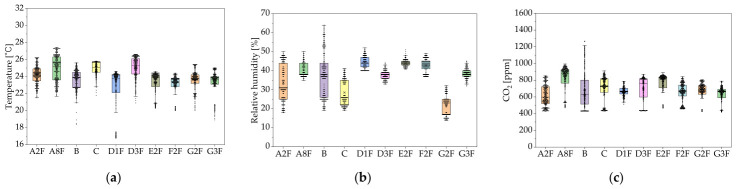
Indoor temperature (**a**), relative humidity (**b**), and CO_2_ concentration (**c**) in each investigated office space during winter.

The distributions of indoor temperature, RH, and CO_2_ concentrations during working hours in summer and winter are shown in Figure 1 and Figure 2, respectively.

The median indoor temperature during summer ranged from 25.2 to 27.4 °C. Although all median values satisfied the maintenance standard specified by the Japanese Act on Maintenance of Sanitation in Buildings (18–28 °C), some measurements in D3F exceeded the upper limit of the standard (28 °C). In winter, the median indoor temperature ranged from 23.4 to 25.3 °C; however, some measurements in D1F fell below the lower limit of the standard (18 °C).

The RH values during summer remained below 70% in all office spaces, meeting the requirements of the Japanese Act on Maintenance of Sanitation in Buildings. In contrast, many winter measurements fell below the lower limit of 40%, indicating prevalent dry indoor conditions during the winter season. Regarding CO_2_ concentration, all measurements met the maintenance standard of 1000 ppm, except for most measurements in Building B during summer and some measurements in Building B during winter.

Overall, indoor temperature and CO_2_ concentration, which is an indicator of ventilation adequacy, generally satisfied the requirements of the Japanese Act on Maintenance of Sanitation in Buildings. However, a marked decrease in RH was observed during winter, resulting in low-humidity conditions in many office spaces.

### 3.2. Indoor and Outdoor FAP Concentrations in Summer and Winter

**Figure 3:**
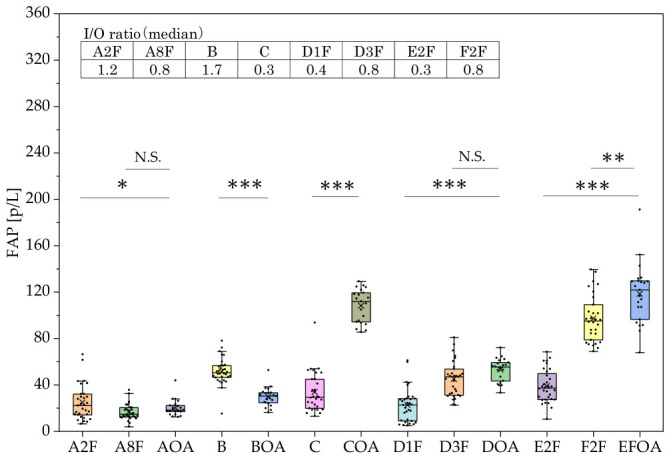
Distribution of indoor and outdoor fluorescent aerosol particle (FAP) concentrations and median indoor-to-outdoor (I/O) ratios in each investigated office space during summer. *: *p* < 0.05, **: *p* < 0.01, ***: *p* < 0.001, N.S. Not Significant.

**Figure 4:**
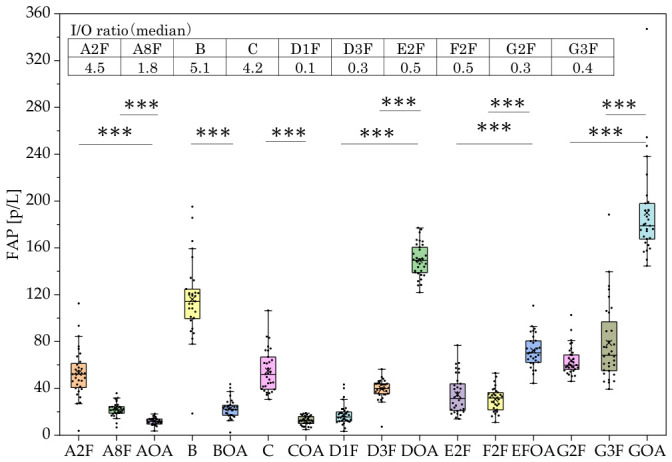
Distribution of indoor and outdoor fluorescent aerosol particle (FAP) concentrations and median indoor-to-outdoor (I/O) ratios in each investigated office space during winter. ***: *p* < 0.001.

Figure 3 and Figure 4 show the distributions of indoor and outdoor FAP concentrations and the corresponding median indoor-to-outdoor (I/O) ratios during summer and winter. Among the indoor measurements conducted during summer, F2F exhibited the highest FAP concentration, with the 75th percentile exceeding 100 p/L (particles L^−1^) and a maximum value of 140 p/L. In contrast, all measurements in the other office spaces were below 100 p/L. During winter, Building B showed the highest indoor FAP concentration, with the 25th percentile exceeding 100 p/L and a maximum value of 195 p/L. For all other office spaces, the 75th percentile remained below 100 p/L.

Seasonal differences in indoor FAP concentrations were assessed using the Wilcoxon signed-rank test. Significant differences were observed in all office spaces except D1F (*p* = 0.142), D3F (*p* = 0.268), and E2F (*p* = 0.256) (all other offices: *p* < 0.001). Indoor FAP concentrations were significantly higher in winter than in summer in A2F, A8F, B, and C. In contrast, F2F exhibited significantly higher FAP concentrations in summer than in winter. Comparisons between indoor and outdoor FAP concentrations revealed significant differences in all office spaces during summer except A8F (*p* = 0.261) and D3F (*p* = 0.067) (A2F: *p* < 0.05, G3F: *p* < 0.01, others: *p* < 0.001). During summer, among the office spaces showing significant differences, indoor FAP concentrations were significantly higher than outdoor concentrations in A2F and B, whereas the opposite trend was observed in the remaining office spaces. During winter, indoor FAP concentrations were significantly higher than outdoor concentrations in all four office spaces in Buildings A–C. In contrast, indoor concentrations were significantly lower than outdoor concentrations in the six office spaces in Buildings D, E, F and G (all *p* < 0.001).

### 3.3. Size-Resolved Distribution of FAP Concentrations

**Figure 5:**
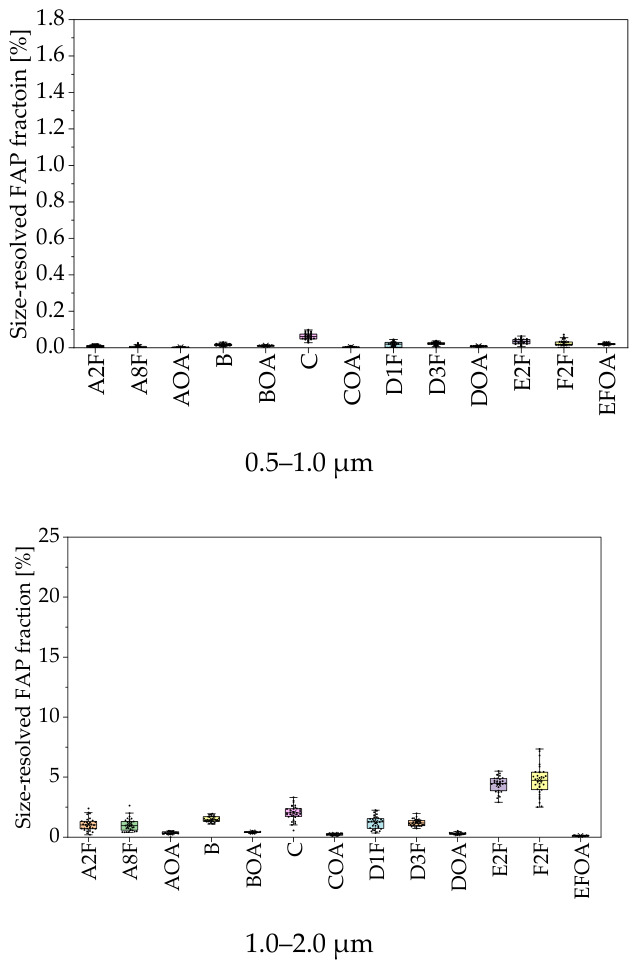

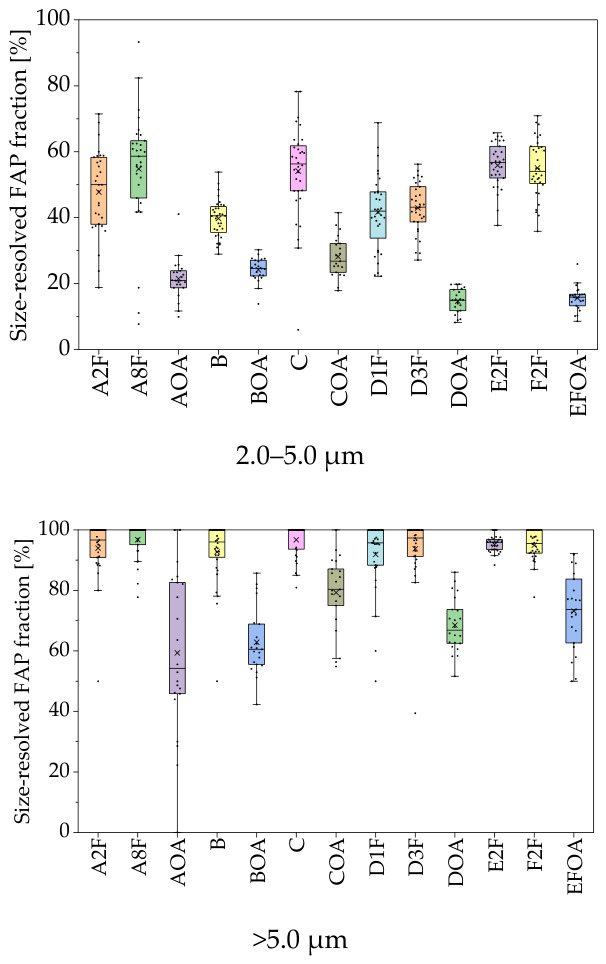
Size-resolved FAP fraction for indoor and outdoor environments in each investigated office space during summer.

**Figure 6:**
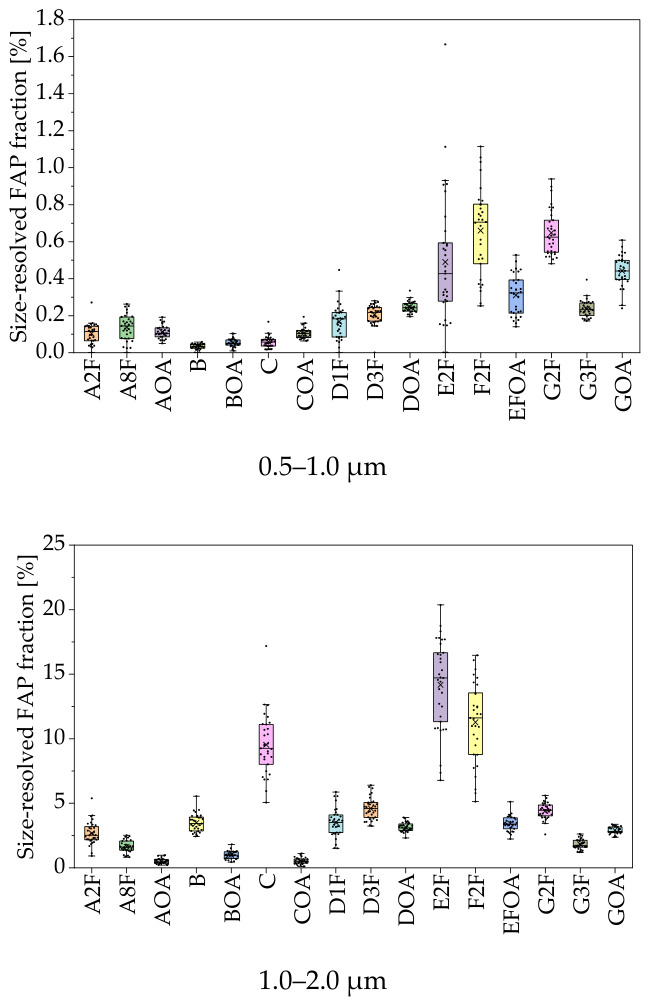

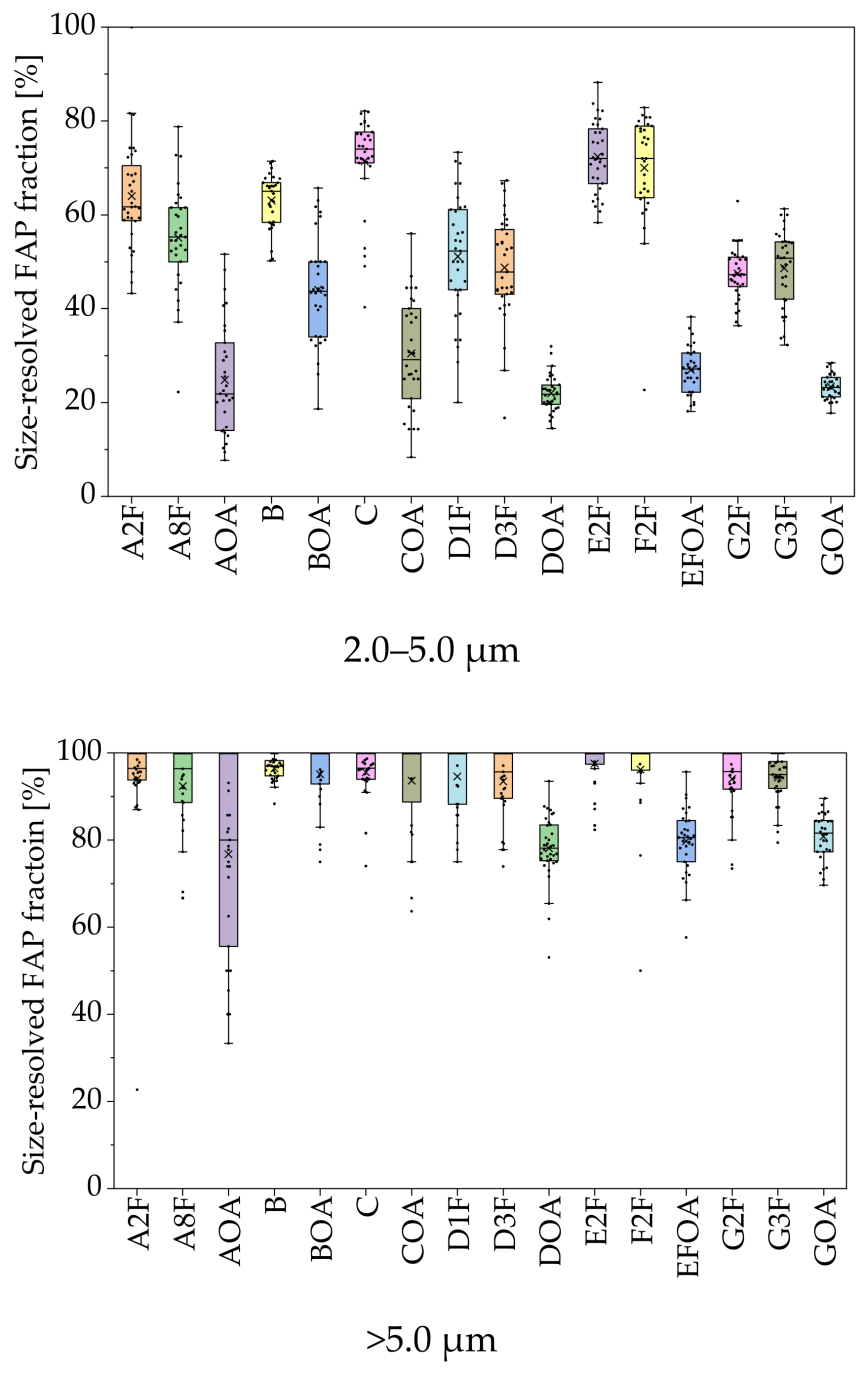
Size-resolved FAP fraction for indoor and outdoor environments in each investigated office space during winter.

The size-resolved FAP fraction was defined as the FAP concentration in each particle-size category divided by the total FAP concentration across all measured particle-size categories. Figure 5 and Figure 6 show the size-resolved FAP fraction (%) for each particle-size category during summer and winter, respectively. Regardless of season, size-resolved FAP fraction increased significantly with particle size in both indoor and outdoor environments. The median size-resolved FAP fraction for each particle-size category is summarized in Table 2.

For indoor environments, the median size-resolved FAP fraction was ≤0.1% for particles smaller than 1.0 μm, ≤9.3% for particles between 1.0 and 2.0 μm, 40.5–74.0% for particles between 2.0 and 5.0 μm, and 96.4–100% for particles larger than 5.0 μm. Corresponding outdoor median values were ≤0.4%, ≤3.4%, 15.0–43.8%, and 54.2–100%, respectively.

Comparison of size-resolved FAP fraction between indoor and outdoor environments showed that, during summer, indoor size-resolved FAP fraction were significantly higher than outdoor values for all particle-size categories larger than 1.0 μm in every office space (*p* < 0.001). In contrast, no consistent indoor–outdoor pattern was observed during winter.

Statistical analyses comparing indoor size-resolved FAP fraction between summer and winter revealed significant differences in many office spaces. However, some office spaces exhibited higher values in summer, whereas others showed higher values in winter, indicating that the seasonal influence on size-resolved FAP fraction varied among buildings.

## 4. Discussion

Occupant activities, ventilation, and environmental conditions have long been recognized as major determinants of indoor bioaerosol concentrations [16,17,18]. The present study investigated the seasonal and size-resolved characteristics of FAPs in office environments across multiple regions of Japan. Overall, indoor temperatures and CO_2_ concentrations generally satisfied the requirements of the Japanese Act on Maintenance of Sanitation in Buildings, indicating adequate thermal conditions and ventilation. In contrast, RH frequently fell below 40% during winter, confirming the prevalence of dry indoor environments in office buildings. Previous studies have suggested that low relative humidity may promote particle resuspension, thereby facilitating the release of settled bioaerosols into the air [15,39]. Although D3F exceeded 28 °C during part of the summer and D1F occasionally fell below 18 °C during winter, no corresponding increase in FAP concentrations was observed at these locations. These findings suggest that the limited temperature deviations observed in this study had less influence on FAP concentrations than other factors such as relative humidity, occupant activities, and building-specific characteristics. Consistent with this mechanism, indoor FAP concentrations were significantly higher in winter than in summer in several office spaces.

These findings suggest that dry indoor conditions may promote the resuspension and prolonged airborne persistence of biological or biologically related particles. However, some office spaces exhibited higher FAP concentrations during summer, indicating that FAP levels are influenced not only by humidity but also by occupant activities, outdoor particle concentrations, and building-specific characteristics. Similar factors influencing indoor bioaerosol concentrations have recently been summarized by Jabeen et al. [40], who reported that indoor bacterial aerosol concentrations are strongly affected by occupant activities and ventilation conditions.

The indoor FAP concentrations observed in this study were generally below 100 p/L and were comparable to those reported previously for university classrooms (10–100 p/L) [41] and residential environments (several to several tens of p/L) [42]. Significant seasonal differences were detected in most office spaces, although the direction and magnitude of these differences varied among buildings. Previous outdoor measurements have shown that FBAPs are typically more abundant during summer, likely reflecting enhanced plant activity and fungal growth [25,43]. Li et al. also reported temporal variations in indoor and outdoor fluorescent biological aerosol particles associated with occupant activities and environmental conditions [44]. In contrast, several offices investigated in the present study exhibited higher FAP concentrations during winter, suggesting that indoor sources can be as important as, or even more important than, outdoor bioaerosol inputs in determining indoor FAP concentrations.

Indoor–outdoor comparisons further supported the existence of multiple FAP sources. Recent atmospheric observations have also shown that long-range transport and outdoor sources substantially contribute to fluorescent aerosol particle concentrations [45]. These findings are consistent with the importance of outdoor infiltration observed in the present study. FAPs are commonly used as indicators of bioaerosol particles; however, they may also originate from non-biological fluorescent materials, including textile fibers, detergent residues containing optical brighteners, and cosmetic particles [30,46]. Consequently, indoor FAP concentrations reflect not only biological emissions but also human activities and occupant-related processes. Previous chamber studies demonstrated that clothing and clothing–skin friction are important sources of coarse fluorescent particles [19], while Yang et al. reported approximately 40% higher FAP emissions from occupants wearing long-sleeved clothing compared with those wearing short-sleeved clothing [39]. Other studies have shown that occupied indoor environments contain substantially higher concentrations of FBAPs than unoccupied environments [41], and that human occupants represent a major source of fluorescent particles in office buildings [47]. Therefore, differences in occupant density, activity level, and clothing characteristics may have contributed to the observed variations in indoor FAP concentrations.

To estimate the relative contributions of indoor and outdoor sources, indoor-to-outdoor (I/O) ratios were evaluated. D, E and F consistently exhibited I/O ratios below unity during both seasons, suggesting a stronger influence of outdoor-derived particles. Differences in HVAC configuration, filtration performance, and outdoor air supply rates may also have contributed to the observed variations among buildings. In contrast, A2F and B exhibited I/O ratios greater than unity in both summer and winter, indicating substantial contributions from indoor sources such as occupant activities and particle resuspension from indoor surfaces. The elevated wintertime I/O ratios observed in these offices may be partially attributable to enhanced resuspension under low-humidity conditions.

The observed I/O ratios should also be interpreted in consideration of the HVAC systems. Although the filtration efficiencies of the AHU filters were unavailable for Buildings B and C, the filters used in the other buildings corresponded approximately to MERV 11–13, which can remove a substantial fraction of particles larger than 1 μm. Consequently, outdoor FAP concentrations are expected to be reduced before supply air enters the indoor environment. Therefore, an I/O ratio greater than unity provides strong evidence for dominant indoor sources of FAPs. Conversely, an I/O ratio below unity should be interpreted with caution because the supply-air concentration is lower than the outdoor concentration due to filtration, and thus the actual indoor concentration relative to the supply air is higher than indicated by the measured I/O ratio. In addition, differences in outdoor air exchange rates among buildings may also influence I/O ratios, although these data were not available in the present study.

Previous investigations have evaluated the contribution of primary biological aerosol particles to airborne particulate matter in indoor and outdoor environments; however, corresponding information for mechanically ventilated office buildings remains scarce [48]. To our knowledge, few studies have quantitatively evaluated the size-resolved contribution of fluorescent particles to total particulate matter in office environments. The most notable finding of this study was the pronounced increase in the size-resolved FAP fraction with increasing particle size. Although the overall proportion of FAPs in total particulate matter was relatively low (0.2–1.7% in summer and 1.1–10.4% in winter), size-resolved analysis revealed a strong dependence on particle diameter. Median indoor size-resolved FAP fraction values were less than 15% for particles smaller than 2.0 μm, increased to 40–74% for particles between 2.0 and 5.0 μm, and exceeded 95% for particles larger than 5.0 μm. Similar trends were observed outdoors. These results indicate that fluorescent particles are strongly associated with the coarse particle fraction.

Individual bacterial cells generally range from approximately 0.5 to 2 μm in diameter, whereas fungal spores and plant-derived particles are often several micrometers or larger [18,43]. In addition, bacterial aggregates and skin flakes can occur in the coarse particle size range [18]. Therefore, the increasing size-resolved FAP fraction ratio with particle size likely reflects the growing contribution of biological particles within the coarse aerosol fraction. The observation that size-resolved FAP fraction approached 100% for particles larger than 5 μm suggests that coarse particles in office environments are predominantly composed of fluorescent biological or biologically related particles. Collectively, these findings indicate that FAPs in office environments are strongly concentrated in the coarse particle fraction and that particles larger than 2.0 μm represent an important component of indoor bioaerosol exposure. These findings suggest that monitoring coarse fluorescent particles may provide a practical indicator for assessing bioaerosol-related indoor environmental quality in office buildings.

Although the size-resolved FAP fraction ratio approached 100% for particles larger than 5.0 μm, the absolute number of coarse particles was relatively low. Therefore, the calculated ratio may be more sensitive to counting fluctuations in this size range. Nevertheless, the consistently high size-resolved FAP fraction observed across multiple office spaces and both seasons suggests that the strong size dependence is unlikely to be solely an artifact of low particle counts.

## 5. Study Limitations

Several limitations should be acknowledged in this study. First, FAPs measured by BAS represent fluorescence-emitting particles and should be interpreted as a proxy for PBAPs rather than direct measurements of biological particles. Fluorescent signals can also originate from non-biological materials such as textile fibers, optical brighteners, and combustion-derived particles, as demonstrated in recent atmospheric observations [49]. Another limitation is that the size-dependent fluorescence response of the BAS was not independently evaluated using standardized fluorescent particles. Although previous calibration confirmed the discrimination between fluorescent and non-fluorescent particles, the influence of particle size on fluorescence detection efficiency remains uncertain. Therefore, quantitative comparisons among particle-size classes should be interpreted with some caution. Future studies should investigate the size-dependent fluorescence response of the BAS using standardized fluorescent particles to further validate quantitative comparisons among particle-size classes. Second, measurements were conducted during representative occupied periods because BAS measurements were performed sequentially across multiple buildings. Consequently, the measurement duration at each site was limited to approximately 30 min, which may not fully capture diurnal variations in indoor bioaerosol concentrations. Third, occupancy levels were not standardized among buildings and may have contributed to the observed variability. Finally, microbial composition was not analyzed in the present study. Future studies combining real-time fluorescence monitoring with microbial culture methods and DNA-based analyses are needed to better identify the sources and characteristics of fluorescent particles in office environments.

## 6. Conclusions

This study investigated the characteristics of FAPs in ten office spaces located in four distinct regions of Japan during summer and winter using a real-time BAS. The major findings are summarized as follows:

1. Indoor FAP concentrations were generally below 100 p/L, although maximum concentrations of 140 p/L in summer and 195 p/L in winter were observed in specific offices.
2. Significant seasonal differences in indoor FAP concentrations were observed in most offices. In several buildings, winter concentrations were significantly higher than summer concentrations, suggesting that low indoor humidity may enhance particle resuspension.
3. Indoor–outdoor comparisons revealed considerable building-to-building variability. Some offices showed I/O ratios greater than 1, indicating substantial indoor sources, whereas others consistently exhibited I/O ratios below 1, suggesting a stronger influence of outdoor particles.
4. The size-resolved FAP fraction increased markedly with particle size. Median indoor size-resolved FAP fraction exceeded 40% for particles between 2.0 and 5.0 μm and reached more than 95% for particles larger than 5.0 μm.
5. The seasonal and indoor–outdoor variations observed among buildings indicate that both indoor activities and outdoor particle infiltration play important roles in determining FAP concentrations in office environments.
6. These findings demonstrate the potential of real-time fluorescence monitoring for identifying seasonal bioaerosol dynamics and supporting indoor environmental quality assessments in office buildings.

Overall, the findings provide new insights into the seasonal behavior and size-dependent characteristics of FAPs in office environments. To our knowledge, this is one of the first studies to characterize seasonal and size-resolved FAP distributions in multiple office buildings across Japan. Real-time fluorescence monitoring using BAS may serve as a useful tool for assessing bioaerosol-related indoor air quality and identifying environments with elevated biological particle loads.

## Figures and Tables

**Table 1:** Overview of the investigated office buildings and their air-conditioning systems.

ID	Location	Year of Construction	Measurement Floor(s)	Office Floor Area (m^2^)	Air Conditioning System
A	Miyazaki City, Miyazaki	2020	2F	540	AHU
		2020	8F	540	
B		1968	3F	225	PAC
C		1990	6F	173	AHU
D	Inzai City, Chiba Prefecture	1993	1F	1195	AHU
		1993	3F	492	Radiant Chilled-Water Panel + FCU
E	Fujisawa City, Kanagawa Prefecture	2006	2F	1555	AHU
F		1996	2F	1305	AHU + FCU
G	Iruma City, Saitama Prefecture	1984	2F	354	Outdoor-Air Processing PAC
		1984	3F	354	

AHU: Air Handling Unit; PAC: Packaged Air Conditioner.

**Table 2:** Median Size-Resolved FAP Fractions (%) by Particle Size in Summer and Winter.

Particle Size Range	Summer (August 2025)		Winter (February 2026)	
	Indoor Air	Outdoor Air	Indoor Air	Outdoor Air
0.5–1.0 μm	0.0–0.1	0.0–0.0	0.0–0.1	0.1–0.4
1.0–2.0 μm	1.0–2.0	0.1–0.4	1.6–9.3	0.5–3.4
2.0–5.0 μm	40.5–58.6	15.0–26.8	55.3–74.0	21.6–43.8
>5.0 μm	96.4–100.0	54.2–80.4	96.4–97.0	78.1–100.0
Total	0.2–1.7	0.1–0.2	1.1–10.4	0.2–2.3

## Data Availability

Data will be made available on request.
